# Sensory evaluation and consumer acceptability of a beverage made from malted and fermented cereal: case of gowe from Benin

**DOI:** 10.1002/fsn3.166

**Published:** 2014-12-02

**Authors:** Laurent Adinsi, Noël H Akissoé, Générose Dalodé-Vieira, Victor B Anihouvi, Geneviève Fliedel, Christian Mestres, Joseph D Hounhouigan

**Affiliations:** 1 Faculté des Sciences Agronomiques, Université d'Abomey-CalaviCotonou, 01 BP 526, Bénin; 2 CIRAD UMR Qualisud TA B-95/1673 rue Jean-François Breton, 34398, Montpellier Cedex 5, France

**Keywords:** Consumer acceptability, gowe, maize, sensory evaluation, sorghum

## Abstract

Sensory profile of gowe beverage was established with 10 gowe samples by 22 semitrained panelists. Besides, consumer study was performed on four representative gowe samples with 141 African ordinary consumers using a modified quantitative descriptive analysis. Gowe samples significantly differed (*P* < 0.05) with respect to all the sensory attributes, except for cereal odor and cereal taste (*P* > 0.05). The principal component analysis plot revealed the effects of raw material and process: Sorghum gowe was differently scored from maize gowe samples (*P* < 0.05). Gowe types from saccharification step (SSaF, SSaSF) evidenced higher scores with respect to fermented odor (41.7) and acidic taste (47.9), while those without saccharification had lower scores of fermented odor and acidic taste, with values of 18.4 and 16.9, respectively. No significant difference was evidenced with respect to the addition of “non malted flour” before or after saccharification. Regarding consumer testing, three distinct patterns of consumer acceptability were observed, which were grouped as “Sugary gowe likers” (63.1% of consumers) followed by “Sugary and saccharified sorghum gowe likers” (20.6%) and “Pure maize gowe dislikers” (16.3%). Irrespective of the consumers cluster, saccharified malted sorghum gowe without sugar was the unique sample scored more than 6 over 9.

## Introduction

In West Africa, particularly in Benin, traditional processors have developed many food processing techniques as a response to environmental constraints and consumers' demand. As far as cereal-based foods are concerned, most of these processes include malting and fermentation steps, which improve not only the sensory quality but also the nutritional quality of the end products (Kazanas and Fields 1981; Chavan et al. 1988).

Gowe is a traditional Beninese product made from malted and nonmalted maize or sorghum flours which are spontaneously fermented and then cooked to give sweet dough (Adinsi et al. 2014). It is consumed as is or after diluting in water often with the addition of sugar. It is produced by small-scale processors and consumed as a thirst quenching and energetic drink. Originally, gowe was popular in the center of Benin (Michodjèhoun-Mestres et al. 2005; Adinsi et al. 2014) but its consumption has spread to other regions of the country, essentially to the main cities. This expansion indeed shows the need for medium-or large-scale commercial production of traditional products for the local and regional market.

A recent survey reported different types of gowe that differ in raw materials and processing technology. This variability resulted from endogenous innovative actions of producers (Adinsi et al. 2014). It appears, in particular, that sorghum and maize are used singly or in combination and that gowe processing still relies on spontaneous fermentation (Michodjèhoun-Mestres et al. 2005; Vieira-Dalodé et al. 2007; Adinsi et al. 2014). The variability in the raw materials and processing methods can be source of variations in quality attributes such as taste, odor, and texture, which need to be described. In addition, gowe quality can vary during selling/storage since gowe is indeed a wet paste wrapped in vegetable leaves, with a short shelf-life (about 2–4 days). Although gowe consumption cuts across all classes of people (Adinsi et al. 2014), no relationship has been established between consumer preference and the sensory attributes of the type of gowe. Little is also known regarding the sensory properties of gowe beverage and their physicochemical characteristics.

This study describes the consumer acceptability and its relationship with the sensory attributes and physicochemical characteristics of gowe. The results of this work are an important background for guiding the development of gowe that fit market demand.

## Materials and Methods

### Experimental samples

White maize grains (*Zea mays*) and red sorghum grains (*Sorghum bicolor* (L.) Moench) were purchased from the international market of Dantokpa (Cotonou, Benin). Five types of gowe were processed by traditional producers using the traditional method (Adinsi et al. 2014) and the raw materials under good hygienic conditions.

### SSaSF: saccharified malted sorghum gowe

Sorghum grain was cleaned and divided into two parts. One part (25%) was soaked, germinated, and sun dried. The resulting malted grain (25%) and raw grain (no-malted) (75%) were milled separately using a plate disk mill. One part of malted sorghum flour (20%) was kneaded with tap water to obtain dough which was left for saccharification at ambient temperature (28–32°C) for 6 h. After saccharification, the remaining malted flour (5%) was mixed with the no-malted flour (5–10%) for preparing a slurry using tap water (ratio of 1/6 [flour/water]). The latter was precooked (60–70°C) and then added to the saccharified dough. The resulted product was mixed with the remaining raw grain flour (65–70%). Sufficient water was added to the mixed dough that undergoes solid-state spontaneous fermentation (12 h). It was then cooked for 45 min.

### SSaF: saccharified malted and no-malted sorghum gowe

The SSaF was a variant of SSaSF except the fact that the malted (25%) and raw grain (75%) flours were mixed at the beginning of the process.

### SF: sorghum gowe

As for SSaF, malted (25%) and raw grain (75%) flours were mixed at the beginning of the process. The difference is that the slurry is directly added to the dough (without the saccharification step) and the mixture was left for spontaneous fermentation (16 h) before cooking.

### MF: maize gowe

Maize gowe was produced as described for SF but sorghum is replaced by maize.

### XF: mix cereal gowe

Mix cereal gowe was produced as described for SF but a mixed flour of 50% of malted sorghum and 50% of raw grain maize (50%) was used. The fermentation duration was in this case 20 h.

### Ethical assessment and consent

Prior to be enlisted in the consumer and descriptive panel, members were briefed about the study to enable them to make an informed decision. Those who agreed to participate had to sign consent forms. Members were free to withdraw from the study at any time.

### Sensory evaluation

Each type of gowe was consumed in two forms: plain (no ingredient added) and diluted with water and the addition of sugar (4.7%, w/w of diluted gowe). The 10 gowe samples (Table1) were scored by a semitrained sensory panel using a modified version of quantitative descriptive analysis since standards were not provided (Meilgaard et al. 2007; Tomlins et al. 2012). The panel was composed of technicians and students from the University of Abomey-Calavi, and employees of private companies (22 panelists). Sessions were conducted at the University of Abomey-Calavi (South Benin) under air conditioned and artificial lighting environment. The panelists were spaced at least 2 m in a booth area to avoid interaction. The panelists were selected for perception of the basic tastes (sweet and sour) and familiarity with the product. Sensory attributes were generated during a preliminary focus group session using gowe samples widely differing in their sensory characteristics. After eliminating similar terms, 13 descriptive terms were generated (Table2). Intensity ratings were scored on a 100 mm unstructured anchored scale with the terms “lowest rating” at the low end and “highest rating” at the high end.

**Table 1:** Gowe samples tested for sensory

Raw material	Processing technology summary	Tested forms	Initials^1^
Sorghum	Malted sorghum (25%) + No-malted sorghum (75%)/saccharification/fermentation/cooking	Plain gowe sorghum	SSaFp
		Diluted sorghum gowe with sugar	SSaFs
	Malted sorghum (25%) + no-malted sorghum (75%)/fermentation/cooking	Plain gowe sorghum	SFp
		Diluted sorghum gowe with sugar	SFs
	Malted sorghum (25%)/saccharification/adding of no-malted sorghum/fermentation/cooking	Plain gowe sorghum	SSaSFp
		Diluted sorghum gowe with sugar	SSaSFs
Maize	Malted maize (25%) + no-malted maize (75%)/fermentation/cooking	Plain gowe maize	MFp
		Diluted maize gowe with sugar	MFs
Mix “sorghum and maize”	Malted sorghum (50%) + no-malted maize (50%)/fermentation/cooking	Plain gowe mix cereals	XFp
		Diluted mix cereals gowe with sugar	XFs

1 Definition of initials: First S, sorghum; M, maize; X, mix “sorghum and maize”; Sa, saccharification; second S, sorghum flour; F, fermentation; p, plain (no ingredient added); s, sugar.

**Table 2:** Descriptors for gowe

Sensory attributes	Description
Brown color	Color characteristic of brown sorghum
White color	Color characteristic of white maize
Concentrated aspect	Related to the difficulty to flow with a high proportion of solid matter
Presence of bran	Related to bran particles in gowe
Grainy	Appearance of small particles
Presence of lumps	Appearance of several agglomerated particles in the liquid
Sweet taste	Taste sensation that is related to sugar
Acidic taste	Taste characteristic of lemon
Cereal taste	Taste characteristic of cereal (taste related to maize or sorghum)
Aftertaste	Sensation after swallowing that looks like abnormal
Cereal odor	Odor characteristic of cereal (aroma related to sorghum or/and maize)
Fermented odor	Aroma typical of fermented alcoholic products
Burnt odor	Odor sensation that looks like abnormal

After panel training, four gowe samples (coded with three-figure random numbers) were evaluated at each session. They were served in random order to each panelist. Panel sessions were conducted until all samples were scored in triplicate within four consecutive days. Gowe samples were freshly prepared every day and kept in a cooled box until serving. The panelists rinsed their mouths with mineral water before testing each sample.

### Consumer acceptance

Consumer acceptance was assessed at two locations in Benin (Cotonou and Abomey-Calavi) on a subsample of four gowe which were reasonably chosen in each cluster and then presented to consumers following a balanced order for each participant. One hundred and forty-one African consumers scored their liking for appearance, taste, and overall liking of gowe using a 9-point hedonic box scale (Meilgaard et al. 2007) from “dislike extremely” to “like extremely.” Each gowe sample (50 mL) was coded with three random numbers and presented simultaneously, but in random order to each consumer.

After testing the products, consumers were interviewed for gathering information on gender, age, occupation, marital status, number of children, education level, type of gowe usually consumed, form of consumption, frequency of consumption, constraint limiting the consumption, place where gowe has been eaten, and period of consumption.

### Physicochemical analyses

The water content of gowe samples was determined as described in AACC 44-15 (1984). The pH was determined using an InoLab digital pH-meter (WTW series 730) calibrated with buffers at pH 4.0 and 7.0 (WTW, Weilheim, Germany). The titratable acidity, expressed as lactic acid equivalent, was performed by titrating 10 g of gowe using 0.1 N NaOH (Merck, Darmstadt, Germany) as described by AACC 02-31.01. The apparent viscosity was determined on diluted gowe using a Rapid Visco Analyser (RVA; Newport Scientific, Narabeen, Australia). Twenty-eight grams of homogeneized sample was heated at 35°C for 3 min with stirring rate of 160 rpm and mean apparent viscosity was recorded.

### Statistical analysis

Analysis of variance (ANOVA), Kruskal–Wallis test, correlation analysis (Pearson), cluster analysis, principal component analysis (PCA), and internal preference mapping were computed using Statistica 7 (StatSoft, Tulsa, OK) and XLSTAT (V 5.2; Addinsoft, Paris, France).

## Results and Discussion

### Sensory profile of gowe

There were significant differences (*P* < 0.001) among the panelists for every sensory attribute, and significant interactions between sensory attributes and panelists (*P* < 0.001) for concentrated aspect, presence of bran, sweet, acidic, and cereal tastes (Table3). Panelists were indeed only briefly trained. Nevertheless, gowe samples significantly differed (*P* < 0.05) with respect to all the sensory attributes, except for cereal odor and cereal taste (*P* > 0.05).

**Table 3:** *P*-values of two-ways of variance of sensory attributes of gowe

Descriptors	Samples	Panelists	Sample × panelists
White color	<0.001	<0.001	0.44
Brown color	<0.001	<0.001	0.98
Concentrated aspect	<0.001	<0.001	<0.001
Presence of bran	<0.001	<0.001	<0.001
Grainy	<0.001	<0.001	0.02
Presence of lumps	<0.001	<0.001	0.56
Sweet taste	<0.001	<0.001	<0.001
Acidic taste	<0.001	<0.001	<0.001
Cereal taste	0.1	<0.001	<0.001
Aftertaste	<0.001	<0.001	0.19
Cereal odor	0.09	<0.001	0.005
Fermented odor	<0.001	<0.001	0.08
Burnt odor	<0.001	<0.001	1.0

Gowe samples were scored less than the medium scale for all sensory attributes except the concentrated aspect (mean score of 56 over 100, Table S1). High variability between gowe samples was observed (standard deviation [SD] > 15) with respect to sweet taste (SD = 19) and acidic taste (SD = 20) color (21 and 24 for white color and brown color, respectively) and concentrated aspect (SD = 23).

Irrespective of raw material, gowe types produced with saccharification step (SSaF, SSaSF) were significantly different from those without saccharification. The former evidenced higher scores with respect to fermented odor (41.7 vs. 18.4) and acidic taste (47.9 vs. 16.9). Concerning plain gowe, no significant (*P* > 0.05) difference was observed for concentrated aspect, presence of lumps, and aftertaste attributes. Sweet and acidic taste attributes of the plain saccharified products (SSaFp, SSaSFp) were, however, significantly scored lower than those without the saccharification step. This observation was not expected since starch hydrolysis is supposed to take place during the saccharification step thus increasing free sugar level (Vieira-Dalodé et al. 2008). The low sweet score of the saccharified product could be due to the production step, particularly the malting step since the diastasic potential of traditional sorghum malt may indeed vary widely, from 55.3 to 152.8 Diastasic Power Units (Kayode et al. 2011). It may also be linked to the fermentation metabolism process; fermentable sugars from SSaFp/SSaSFp would have been used by the microorganisms *Lactobacillus* spp. that will generate lactic acid (Vieira-Dalodé et al. 2008) and yeasts that produce CO_2_ and alcohol. Low acidic taste of SSaF and SSaSF could result from the particular development of yeasts and/or to the lower duration (12 h) of the fermentation step. As expected, the diluted gowe with water and addition of sugar were scored sweeter and less acidic, suggesting that sugar addition masked acid perception.

**Figure 1:**
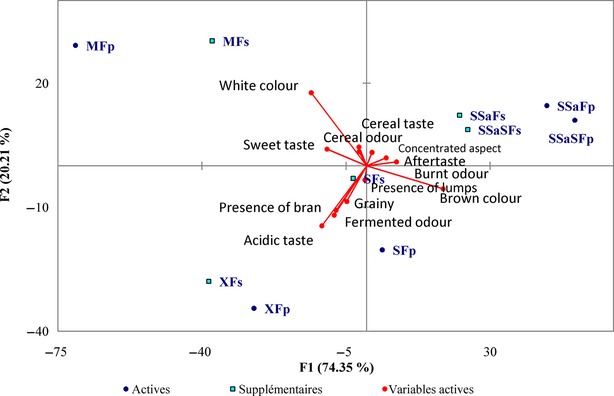
Principal component analysis (PCA) on gowe and sensory descriptors. SSaSFs, saccharified malted sorghum gowe with sugar; SSaSFp, plain saccharified malted sorghum gowe; SSaFs, saccharified malted and no-malted sorghum with sugar; SSaFp, plain saccharified malted and no-malted sorghum; MFs, maize gowe with sugar; MFp, plain maize gowe; SFs, sorghum gowe with sugar; SFp, plain sorghum gowe; XFs, mix cereal gowe with sugar; XFp, plain mix cereal gowe.

A PCA was performed on panel mean sensory attributes. The first two principal components (Fig.1) accounted for 94.6% of the variance of the experimental data. PC1 (74.4% of total variation) was mainly linked to color attributes (brown color plotted opposite to white color) and acidic taste. For PC2, the attributes spanned from white color, acidic taste, fermented odor, and presence of bran. The PCA clearly revealed the effects of raw material and process. Sorghum-based gowe were plotted on the right-hand side of the plan together with brown color, whereas maize-based and mixed gowe were plotted on the left, with white color attribute. Red sorghum is indeed used for preparing gowe which imparts a brownish color to the product whereas white maize is used for MF. In addition, the saccharified samples were grouped alone in the first quarter of the plan with indeed a browner color and less acidic taste. Plain gowe were not, however, clearly separated from diluted and sugar-added gowe.

Hierarchical cluster analysis (Ward's method) evidenced four groups of gowe (Table4). The first cluster (maize gowe with sugar [MFs] and plain maize gowe [MFp]) was composed of pure MF with higher significant scores of white color, cereal odor (48 vs. 39–41 for the other clusters), sweet taste, and cereal taste (46 against 38–39). For this cluster, it seemed that the addition of sugar did not affect the sensorial perception. The second cluster included all sorghum gowe with sugar (SSaFs, SSaSFs, and SFs). It differed from cluster 1 by higher score for brown color but lower score for acidic taste (21 vs. 40 and 50 for clusters 1 and 4, respectively). This cluster was similar to cluster 1 for sweet taste score (49 and 50 for clusters 2 and 1, respectively). The third cluster was saccharified sorghum gowe without ingredient addition (SSaFp and SSaSFp). It only differed from the second cluster by a lower sugary taste. The composition of Clusters 2 and 3 revealed that the addition of nonmalted flour before or after saccharification gave a similar gowe. The last cluster included gowe made from mix “sorghum and maize” (XFs, XFp) and plain sorghum gowe (SFp). It was scored with high fermented odor and acidic taste (59 vs. 18–40). The sensory attributes, for example, concentrated aspect, and burnt odor were not related strongly to any of the clusters.

**Table 4:** Cluster analysis, sensory, and acceptability scores of the different types of gowe

Cluster	1	2	3	4
	MFp	SSaFs	SSaFp	XFp
	MFs	SSaSFs	SSaSFp	SFp
		SFs		XFs
Selected sensory attributes				
Brown color	13a	58bc	75c	43b
Cereal odor	48a	39b	41b	39b
Fermented odor	36ab	23ac	18c	49b
Sweet taste	50a	49a	6b	27c
Acidic taste	40ab	21a	18a	59b
Cereal taste	46a	39b	38b	40b
Mean overall acceptability scores				
Average	5.8b	6.6c	3.8a	6.3bc
SD	2.1	1.6	2.0	1.6

### Consumer acceptability of gowe

We selected four samples, one per cluster, for the consumer acceptability test (Table4). The three gowe with sugar were on average acceptable with mean scores over 5 (neither like, nor dislike); only plain (no ingredient added) sorghum gowe (SSaSFp) scored below 5. The most liked was the saccharified malted SFs (mean score of 6.6), whereas MF with sugar (5.8) was in the intermediate position.

**Figure 2:**
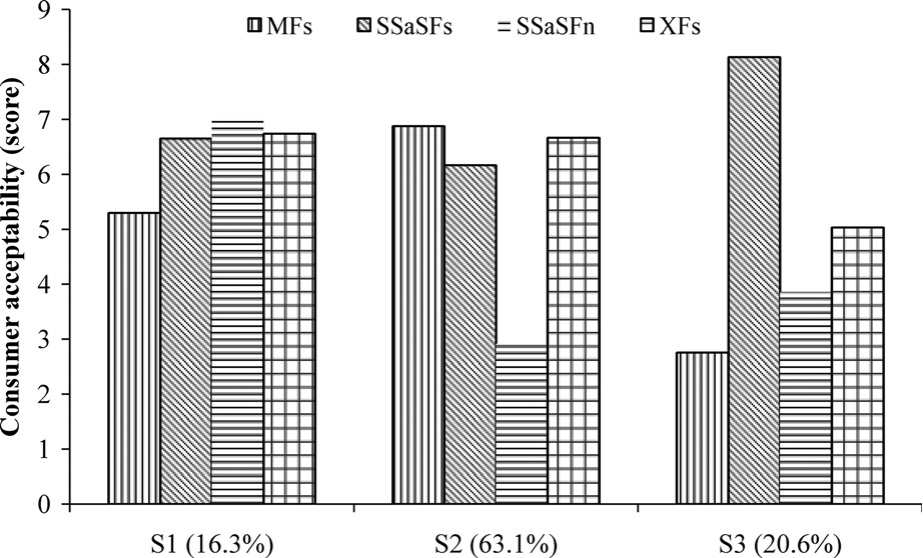
Mean consumer acceptability of gowe by consumer segment. Acceptability was rated on a 9-point scale from 1 = disklike extremely, to 9 = like extremely. SSaSFs, saccharified malted sorghum gowe with sugar; SSaSFp, plain saccharified malted sorghum gowe; MFs, maize gowe with sugar; XFs, mix cereal gowe with sugar; S1,S2,S3, groups.

Hierarchical cluster analysis (Ward's method) indicated that consumers were clustered into three groups as illustrated in Figure2. The largest consumer group 2 gathered 63.1% of consumers, followed by group 3 (20.6%), and group 1 (16.3%). Those in the largest group 2 liked diluted and sugary gowe regardless of the raw material and the technology used and disliked plain gowe. This consumers group could be named “Sugary gowe likers.” Consumers of group 3 only liked diluted gowe from sorghum. Consumers in the smallest group 1 gave high acceptability scores to sorghum gowe samples but the lowest score to the MF. Only saccharified malted sorghum gowe sample with sugar was scored more than a score of 6 in all consumer groups.

**Figure 3:**
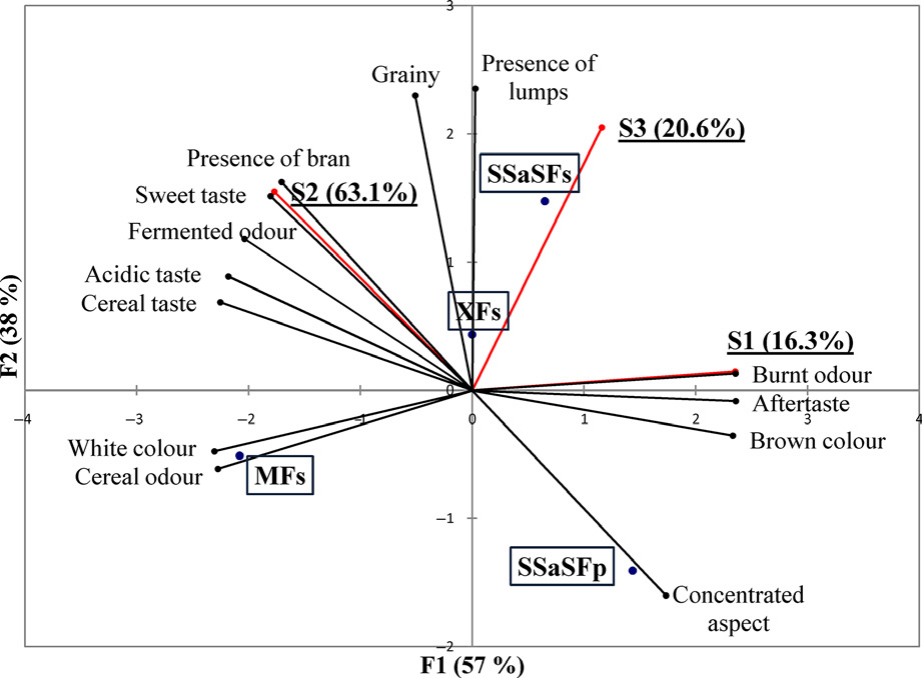
Internal preference mapping relating consumer acceptability (three segments) with sensory attributes by a sensory panel. S1, S2, S3, groups; SSaSFs, saccharified malted sorghum gowe with sugar; SSaSFp, plain saccharified malted sorghum gowe; MFs, maize gowe with sugar; XFs, mix cereal gowe with sugar; Red lines, active variable in PCA; Bold lines, passive variable in PCA.

Internal preference mapping was used to relate the sensory attributes generated by the sensory panel to the mean acceptability of the consumer groups (Fig.3). A PCA was performed on the consumer acceptability score variables with the sensory scores as supplementary or passive variables. The largest consumer group 2 was plotted opposite to plain gowe and concentrated aspect. Acceptability of group 2 was indeed highly negatively correlated with concentrated aspect (Fig.3); these consumers indeed seemed to prefer lighter gowe, even among the diluted ones. Other attributes related to group 2, particulary sweet taste, cereal taste and the presence of bran are linked to the maize gowe. One cannot precisely define what drives the preference of this group of consumers, but these consumers will clearly prefer light and sweet gowe. They will accept gowe from maize or from sorghum and will not reject acidic taste. Consumers in group 3 were plotted close to dilute gowe from sorghum. Disliking plain and maize-based gowe, they were plotted opposite to white color and concentrated aspect. The smallest consumer group 1 who liked every sorghum-based gowe was plotted opposite to pure MF and white color; group 1 acceptability was indeed highly correlated with white color (Fig.3). Other attributes did not seem clearly correlated with the acceptability for this group of consumers and not any tested product appeared close to group 1 acceptability. This means that the ideal product for these consumers was not tested; other experiments are thus necessary to determine the desirable product of these consumers that will prefer gowe from sorghum, contrary to group 2.

Regarding demographic differences between clusters, the Kruskal–Wallis test indicated that the segments differed significantly with respect to marital status (*P* = 0.08) and education level (*P* = 0.03) (Table5); consumers of group 1 appeared more educated. In contrast, they did not differ significantly (*P* > 0.1) with respect to age, gender, occupation, and economic situation. Concerning consumers' attitudes to buy gowe, most consumers (75.3–96.6%) used to buy sorghum gowe, particularly diluted with sugar and/or milk addition. In accordance with their preference, consumers of group 3 never buy MF. For the three clusters, gowe is consumed at home (87.0–93.1%) mainly during the hot period (50.0–61.5%). The frequency of consumption was quite low with almost half of the population consuming gowe once a month. The low frequency of consumption in south Benin is probably related to the difficulty to buy and the trustworthy absence in quality. Indeed, Adinsi et al. (2014) reported that gowe is consumed two to three times per week in traditional/ancestral area of production (center Benin).

**Table 5:** Demographic differences and consumer attitudes to gowe with respect to division cluster

	Segment 1 (16.3%)	Segment 2 (63.1%)	Segment 3 (20.6%)	Kruskal–Wallis test (*P*-values)
Age (years)	30	32	34	0.35
Gender (%)				
Male	69.6	67.4	51.7	0.27
Female	30.4	32.6	48.3	
Marital status (%)				
Married	47.8	52.8	75.0	0.08*
Unmarried	52.2	43.8	25.0	
Education level (%)				
Education more than primary school	95.7	79.3	72.4	0.03*
Occupation (%)				
Civil service	34.8	22.0	39.3	0.5
Housewife	0.0	2.4	0.0	
Artisanship	0.0	20.7	21.4	
Traders	8.7	14.6	10.7	
Student	43.5	24.4	10.7	
Private company employee	13.0	15.9	17.9	
Economic situation (%)				
Bicycle	8.7	2.4	0.0	0.33
Motorbike	69.6	55.0	65.5	0.38
Car	21.7	25.8	17.2	0.61
TV	91.3	79.8	82.8	0.49
House	43.5	30.3	37.9	0.47
Frigo	39.1	29.2	3.4	0.65
Type of gowe purchase (%)				
Sorghum gowe	82.6	75.3	96.6	0.05*
Maize gowe	13.0	15.7	0.0	
“Sorghum and maize” gowe	4.4	9.0	3.4	
Form in which gowe is consumed (%)				
Gowe with water and sugar	77.3	53.9	69.0	0.25
Gowe with water, sugar, and milk	18.2	38.2	17.2	
Nature gowe or gowe with water	4.5	7.9	13.8	
Frequency of consumption (%)				
Consume more than once by month	39.1	52.3	61.7	0.53
Rarely	60.9	47.7	48.3	
Problems with gowe following consumption (%)				
Do not find the good quality in Cotonou	39.1	41.5	27.6	0.56
Availability (place of sale)	60.9	58.5	72.4	0.33
Consumption place (%)				
At home	87.0	92.3	93.1	0.78
At market	13.0	7.7	6.9	
Consumption period (%)				
Hot period	54.5	61.5	50.0	0.8
All period	45.5	38.5	50.0	

* Significant at 10% level.

### Correlations between sensory attributes and physicochemical characteristics

**Figure 4:**
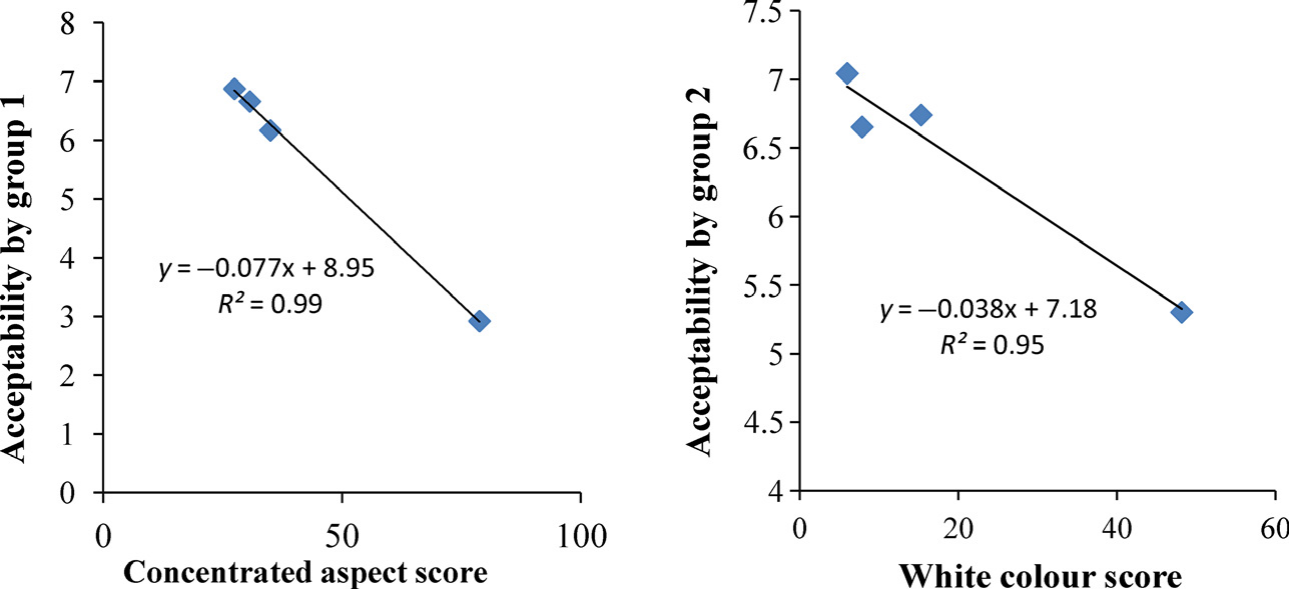
Relationships between sensory attributes, global acceptability, and physicochemical characteristics of gowe.

**Figure 5:**
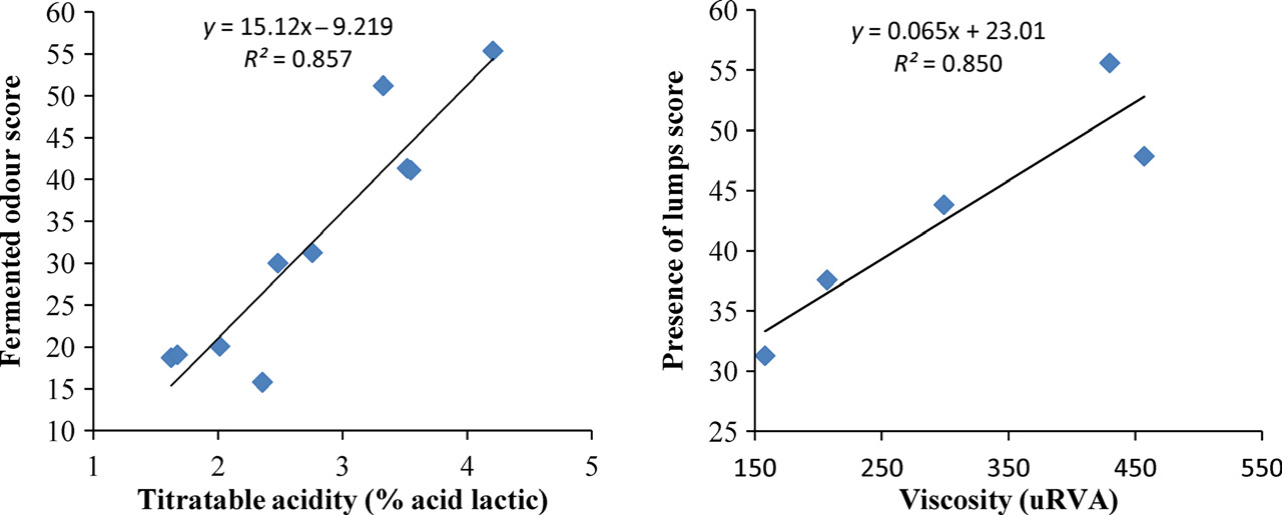
Relationship between physicochemical properties and sensory characteristics of Gowe.

The pH of gowe ranged between 3.7 and 4.6 and the titratable acidity varied from 1.7 to 4.2 (% lactic acid).The dry matter of plain gowe varied between 17.0 and 20.6 and that of diluted and sugary gowe from 13.6 to 15.5. The apparent viscosity of the latter ranged between 158 and 457 uRVA (Table6).These values were in the range of those measured on traditional gowe (Michodjehoun 2000). The saccharified samples presented the lowest titratable acidity and the highest pH (4.3–4.6), in agreement with the lowest acidity scores given by the panel. At the opposite, mixed and MF evidenced the highest titratable acidity and were evaluated accordingly as more acidic by the panel. Concerning consumer testing, acceptability is dependent of sensory attributes while acceptability of groups 1 and 2 was negatively correlated with white color (*r* = −0.97) and concentrated aspect (*r* = −0.99) scores (Fig.4). For the 10 samples of sensory evaluation, titratable acidity was indeed positively and highly correlated (*P* < 0.05) with fermented odor (*r* = 0.93) and acidic taste (*r* = 0.97; Fig.5).

**Table 6:** Physicochemical characteristics of different types of gowe

Samples	pH	Titratable acidity (% lactic acid)	Dry matter (% wet basis)	Viscosity (uRVA)
MFp	3.7	3.5	20.6	
SFp	3.7	3.5	18.2	
SSaFp	4.4	2.0	17.0	
SSaSFp	4.3	2.4	18.8	
XFp	3.8	4.2	17.5	
MFs	4.1	2.5	15.5	207
SFs	3.9	2.8	14.3	299
SSaFs	4.6	1.6	13.8	457
SSaSFs	4.5	1.7	15.2	430
XFs	3.9	3.3	13.6	158

For the texture of the five diluted gowe, no significant correlation was observed between dry matter content and the texture attributes (concentrated aspect, presence of bran, grainy aspect, presence of lumps). Measured viscosity was in addition not correlated with dry matter content; the saccharified gowe presented, in particular, the highest viscosities with low dry matter content for SSaFs (Table6). The high viscosities might be more linked to the starch degradation level, due to malt amylase activity, than to dry matter and starch content itself. Saccharified gowe were judged as the least sugary which indicated a low-starch degradation level. Measured viscosity was, however, significantly correlated with concentrated aspect (*r* = 0.83), presence of bran (−0.86), and presence of lumps (*r* = 0.92; Fig.5). As expected, it appears more difficult to prepare a smooth and even gowe when its viscosity is too high. This confirmed the crucial role of the malt quality on the final quality of the gowe. If the malt is of poor quality, starch degradation during gowe preparation is low and the texture (high viscosity, presence of lumps) is inadequate, particularly for the largest consumer group 2; the sugary taste may be in addition too low.

### Implication for upgrading gowe

Saccharified malted sorghum gowe with sugar (SSaSFs) was accepted by the three consumer segments with high acceptance score (over 6). It looks like a consensual gowe and any barrier for its commercialization for African consumers does not exist. Accordingly, sorghum gowe is more popular in the market than the other types of gowe, and 80.9% of the consumers interviewed commonly consumed this type of gowe. For consumers of group 2, the ideal gowe should have a light texture, without any lump, that is, with a measured viscosity of less than 300 RVU and could be moderately acidic, that is, with a titratable acidity by 2–3% (these values are those of the most acceptable gowe for this group of consumers). It should be noticed that these consumers may also be interested in preparing gowe from maize or from a mixture of sorghum and maize. This study, and particularly consumer segmentation, thus allows to define two types of ideal gowe with common acidity and viscosity specifications but varying in raw material: consensual and traditional one from sorghum and one almost new gowe from maize.

Concerning the processing conditions, the malting step seems a critical point as its success will impart to the product the light texture and sweet taste expected by consumers. The fermentation step can be adapted to control the preferred acidity level.

All this information will be used to reengineer the process to get one or two products corresponding to the demands of African consumers. This will help enhancing the market of this type of traditional products in urban areas that is for the moment hampered by the poor quality and lack of availability of traditional gowe.

## Supporting Information

Table S1. Means and standard deviation for sensory testing with respect to gowe and sensory panelists.
