# Supplementary material for: Sensory evaluation and consumer acceptability of a beverage made from malted and fermented cereal: case of gowe from Benin
Source: Food Sci Nutr. 2014 Dec 2;3(1):1–9. doi: 10.1002/fsn3.166 (PMC4304555; doi:10.1002/fsn3.166)
Supplement: Supplementary file 1 — Table S1. Means and standard deviation for sensory testing with respect to gowe and sensory panelists. [file fsn30003-0001-sd1.doc]

| Descriptors | SSaFp | SSaSFp | SFp | MFp | XFp | SSaFs | | | SSaSFs | SFs | | MFs | | XFs | SD |
| --- | --- | --- | --- | --- | --- | --- | --- | --- | --- | --- | --- | --- | --- | --- | --- |
| White color | 6.4a | 6.0a | 6.3a | 67.0b | 12.0a | 7.5a | | | 7.9a | 12.0a | | 49.5b | | 16.3a | 21.4 |
| Brown color | 72.9bd | 77.0d | 63.0ab | 3.1e | 35.6c | 60.8ab | | | 63.4ab | 50.8a | | 22.3c | | 29.3c | 24.2 |
| Concentrated aspect | 82,5a | 78.8a | 76.5a | 77.4a | 71.3a | 44.3c | | | 35.0bc | 34.4bc | | 26.8b | | 30.5bc | 23.1 |
| Presence of bran | 19.7a | 20.7a | 38.0bc | 42.0cd | 56.4d | |  | 22.0ab | 26.8abc | 36.8abc |  | | 33.3abc | 56.7d | 13.5 |
| Grainy | 19.1a | 22.0a | 31.1ab | 33.4abc | 47.2bc | |  | 25.2a | 33.5abc | 35.9abc |  | | 28.0a | 49.1c | 9.8 |
| Presence of lumps | 24.2a | 29.8ab | 32.2abc | 27.3a | 32.5abc | 47.5cd | | | 55.6d | 43.2bcd | 37.1abc | | | 37.1ab | 9.9 |
| Sweet taste | 5.3a | 6.0a | 29.0b | 47.6cd | 16.7a | 38.8bc | | | 47.4cd | 60.7e | 53.7de | | | 34.7b | 19.4 |
| Acidic taste | 14.8a | 21.5a | 57.4b | 54.0b | 62.9b | 15.0a | | | 16.2a | 30.7a | 25.8a | | | 55.8b | 19.7 |
| Cereal taste | 38.5a | 37.6a | 39.9a | 47.4a | 36.1a | 38.3a | | | 40.9a | 37.0a | 46.3a | | | 37.0a | 3.7 |
| Aftertaste | 41.5ab | 53.4b | 40.5ab | 31.5a | 32.6a | 40.5ab | | | 52.9b | 45.5ab | 33.6a | | | 29.8a | 8.6 |
| Cereal odor | 41.9a | 40.0a | 37.3a | 50.2a | 38.7a | 39.1a | | | 39.9a | 38.8a | 47.3a | | | 41.0a | 3.9 |
| Fermented odor | 20.1a | 15.8a | 41.1bc | 41.3bc | 55.3c | 19.0a | | | 19.1a | 31.3ab | 31.1ab | | | 51.3c | 14.3 |
| Burnt odor | 29.8ab | 49.6d | 30.5a | 14.0c | 20.2abc | 28.7ab | | | 51.4d | 30.0a | 19.3abc | | | 15.2bc | 13.0 |

Table 1- supplemented material: Means and standard deviation for sensory testing with respect to Gowe and sensory panelists

SD : Standard deviation between samples
